# Impulsivity is longitudinally associated with healthy and unhealthy dietary patterns in individuals with overweight or obesity and metabolic syndrome within the framework of the PREDIMED-Plus trial

**DOI:** 10.1186/s12966-022-01335-8

**Published:** 2022-08-08

**Authors:** Carlos Gómez-Martínez, Nancy Babio, Jordi Júlvez, Stephanie K. Nishi, Fernando Fernández-Aranda, Miguel Ángel Martínez-González, Aida Cuenca-Royo, Rebeca Fernández, Susana Jiménez-Murcia, Rafael de la Torre, Xavier Pintó, Mirjam Bloemendaal, Montse Fitó, Dolores Corella, Alejandro Arias, Jordi Salas-Salvadó

**Affiliations:** 1 Universitat Rovira i Virgili, Departament de Bioquímica i Biotecnologia, Unitat de Nutrició Humana, Reus, Spain; 2 Institut d’Investigació Sanitària Pere Virgili (IISPV), Hospital Universitari Sant Joan de Reus, Reus, Spain; 3 Centro de Investigación Biomédica en Red de Fisiopatología de la Obesidad y Nutrición (CIBEROBN), Instituto de Salud Carlos III (ISCIII), Madrid, Spain; 4 Institut d’Investigació Sanitària Pere Virgili (IISPV), Clinical and Epidemiological Neuroscience Group (NeuroÈpia), Reus, Spain; 5 Toronto 3D (Diet, Digestive Tract and Disease) Knowledge Synthesis and Clinical Trials Unit, Toronto, ON Canada; 6 Clinical Nutrition and Risk Factor Modification Centre, St. Michael’s Hospital, Unity Health Toronto, Toronto, ON Canada; 7 Department of Psychiatry, School of Medicine and Health Sciences, University Hospital Bellvitge-IDIBELL and Department of Clinical Sciences, University of Barcelona, Barcelona, Spain; 8 Department of Preventive Medicine and Public Health. IdISNA, University of Navarra, Pamplona, Spain; 9 Integrative Pharmacology and Systems Neurosciences Research Group, Neurosciences Research Program, Hospital del Mar Medical Research Institute (IMIM), Barcelona, Spain; 10 Department of Preventive Medicine, University of Valencia, Valencia, Spain; 11 Faculty of Experimental and Health Sciences, Universitat Pompeu Fabra (UPF), Barcelona, Spain; 12 Lipids and Vascular Risk Unit, Internal Medicine, Hospital Universitario de Bellvitge-IBIDELL, Hospitalet de Llobregat, Barcelona, Spain; 13 Universitat de Barcelona, Barcelona, Spain; 14 Department of Human Genetics, Donders Institute for Brain, Cognition and Behaviour, Radboud University Medical Center, Nijmegen, The Netherlands; 15 Department of Psychiatry, Donders Institute for Brain, Cognition and Behaviour, Radboud University Medical Center, Nijmegen, The Netherlands

**Keywords:** Alternative Healthy Eating Index, DASH diet, Dietary patterns, Mediterranean diet, MIND diet, Planetary Health Diet, Plant-based diet, Portfolio diet, Impulsivity

## Abstract

**Background:**

Few studies have analyzed the associations between impulsivity and dietary patterns. Some of them have shown a cross-sectional inverse relationship between impulsivity and healthy diet scores, whereas others reported a positive association with unhealthy dietary assessments. We aimed to examine longitudinal associations of impulsivity trait with adherence to healthy and unhealthy dietary patterns in older participants at high risk of cardiovascular disease over 3 years of follow-up.

**Methods:**

A 3-year prospective cohort analysis within the PREDIMED-Plus-Cognition study conducted in 4 PREDIMED-Plus study centers was performed. The PREDIMED-Plus study aimed to test the beneficial effect of a lifestyle intervention on the primary prevention of cardiovascular disease. The participants with overweight or obesity and metabolic syndrome included in the present study (*n* = 462; mean age of 65.3 years; 51.5% female) completed both the UPPS-P Impulsive Behavior Scale (range: 0–236 points) and the 143-item Food Frequency Questionnaire at baseline, 1-year and 3-years of follow-up. Ten diet scores assessing healthy and unhealthy dietary patterns were evaluated. Linear mixed models were performed adjusting by several confounders to study the longitudinal associations between impulsivity trait and adherence to dietary pattern scores over 3 years of follow-up (also assessing interactions by sex, age, and intervention group).

**Results:**

Impulsivity were negatively associated with adherence to the Healthy Plant-Based [β = -0.92 (95%CI -1.67, -0.16)], Mediterranean [β = -0.43 (95%CI -0.79, -0.07)], Energy-Restricted Mediterranean [β = -0.76 (95%CI -1.16, -0.37)], Alternative Healthy Eating Index [β = -0.88 (95%CI -1.52, -0.23)], Portfolio [β = -0.57 (95%CI -0.91, -0.22)], and DASH [β = -0.50 (95%CI -0.79, -0.22)] diet scores over 3 years of follow-up, whereas impulsivity was positively related with adherence to the unhealthy Western diet [β = 1.59 (95%CI 0.59, 2.58)] over time. An interaction by intervention group was found, with those participants in the intervention group with high impulsivity levels having lower adherence to several healthy dietary patterns.

**Conclusions:**

Heightened impulsivity was longitudinally associated with lower adherence to healthy dietary patterns and higher adherence to the Western diet over 3 years of follow-up. Furthermore, nutritional intervention programs should consider impulsivity as a relevant factor for the intervention success.

**Trial registration:**

Name of registry: Effect of an energy-restricted Mediterranean diet, physical activity and behavioral intervention on the primary prevention of cardiovascular disease. Trial registration number: ISRCTN 89,898,870. Date of registration: 05/28/2014.

**Supplementary Information:**

The online version contains supplementary material available at 10.1186/s12966-022-01335-8.

## Introduction

Excessive food consumption and weight gain are recognized public health problems, with obesity prevalence being a global epidemic which has tripled since 1975 [1]. Psychological characteristics, such as psychological traits, are considered relevant factors in regard to food choice and dietary intake throughout the lifespan [2–4]. Impulsivity is one psychological trait that has been proposed as being an important factor conditioning eating behaviors, and therefore weight gain or weight loss, and the risk of obesity [5–8].

Personality traits are relatively stable characteristics of individuals, yet can present as a spectrum of possible behaviors given they may be affected by situational cues, specific environmental and social pressures can induce some extreme behaviors across the spectrum of psychological traits [9]. These psychological traits have been recognized as important predictors for health related-outcomes [10]. For example, the impulsivity trait has been proposed as a key factor in successful weight loss in people with extreme obesity who are candidates for bariatric surgery [11]. Impulsivity is defined as “a predisposition toward rapid unplanned reactions to internal or external stimuli without regard to the negative consequences of these reactions to the impulsive individuals or to others” [12]. Impulsivity can affect different aspects of a persons’ life and may be determined by both the genetic background and an individuals’ development [13, 14]. Impulsivity predisposes individuals to respond to their emotional urges, to lack premeditation and planning, and have sensation seeking [15].

Impulsivity has also been related to difficulties delaying immediate rewards [16–18]. In relation to dietary assessments, few cross-sectional studies have suggested that low impulsivity levels are associated with higher adherence to healthy dietary assessments [3, 5]. Specifically, lower impulsivity has also been related with greater control of food intake and body weight [3], and less emotional eating. Furthermore, in comparison to restrained and non-restrained dieters, impulsivity has been postulated to play a role in modifying the participants’ capacity to adhere to an specific diet, which could lead to overeating [6, 7]. Greater impulsivity has also been found to be positively associated with total energy intake, as well as an increased consumption of saturated fats, sugars, appetizers and snacks [5, 19]. Recently, a French study reported that participants with higher levels of impulsivity exhibited a reduced capacity to follow the French nutritional guidelines, assessed by an a priori healthy dietary pattern quality score [5].

To date, limited research has been conducted evaluating the association between impulsivity and dietary assessments, with only cross-sectional evidence being available [3, 5–7]. Additionally, to our knowledge, only one of these aforementioned studies involved analysis of a dietary pattern score [5]. The PREDIMED-Plus-Cognition study presents an opportunity to fill this knowledge gap enabling a longitudinal assessment of impulsivity trait and dietary patterns using repeated measures of impulsivity and diet consumption for a follow-up period of 3 years.

Therefore, the aim of our study was to evaluate longitudinal associations between impulsivity levels and adherence to different dietary pattern scores in an older senior population at high cardiovascular risk. We hypothesized that higher impulsivity trait would be associated with lower adherence to healthy dietary patterns and higher with unhealthy dietary patterns.

## Methods

### Study design

The present work provides an observational prospective cohort design using baseline, 1-year and 3-year follow-up data of the PREDIMED-Plus-Cognition population, a sub-study conducted within the PREDIMED-plus (in Spanish: PREvención con DIeta MEDiterránea) framework. The PREDIMED-Plus study is an ongoing 6-year multicenter, randomized, parallel-group clinical trial conducted in Spain for the primary prevention of cardiovascular disease. Participants were recruited between September 2013 and December 2016 and randomly allocated in a 1:1 ratio to either the intervention or control group, using a centrally controlled, computer-generated random-number internet-based system with stratification by center, sex, and age (< 65, 65–70, and > 70 years). Couples sharing the same household were randomized as pairs, using the couple as a randomization unit. Participants in the intervention group received intensive training to adhere to an energy-reduced Mediterranean diet together with physical activity promotion and behavioral support aimed to achieve and maintain weight loss. Participants in the intervention group were followed 3 times/month (an individual motivational interview, a telephone call, and a group session). Participants in the control group received nutritional educational sessions every 6 months (an individual visit and a group session) to follow a non-caloric reduced Mediterranean diet using the same PREDIMED study approach [20]. No specific advice for increasing physical activity or weight loss was provided to the control group. All participants received free extra virgin olive oil (1 L/month) to reinforce their adherence to the Mediterranean diet. The study protocol has been comprehensively described elsewhere [21, 22], and can be found at http://www.predimedplus.com. The trial was registered at the International Standard Randomized Controlled Trial registry (http://www.isrctn.com/ISRCTN89898870).

### Study population

Eligible participants were aged between 55 and 75 years old with overweight or obesity (27 ≤ BMI < 40 kg/m^2^) meeting at least 3 criteria for metabolic syndrome [23]. From the original PREDIMED-Plus population (*n* = 6,874), the present investigation refers to the PREDIMED-Plus-Cognition study subset of participants (*n* = 487) randomized in the following recruitment centers: Universitat Rovira i Virgili, Universidad de Valencia, Institut Hospital del Mar d’Investigacions Mèdiques-IMIM and Bellvitge University Hospital-IDIBELL. Exclusion criteria are reported elsewhere [21]. For the present analysis, we excluded 25 participants with implausible energy intake values (≤ 800 or ≥ 4000 kcal/d for males; ≤ 500 or ≥ 3500 kcal/d for females) [24], resulting in a total population of 462 participants for the analysis. The flow-chart of the studied participants is shown in Supplemental Fig. 1 [see Additional file 1].

**Fig. 1:**
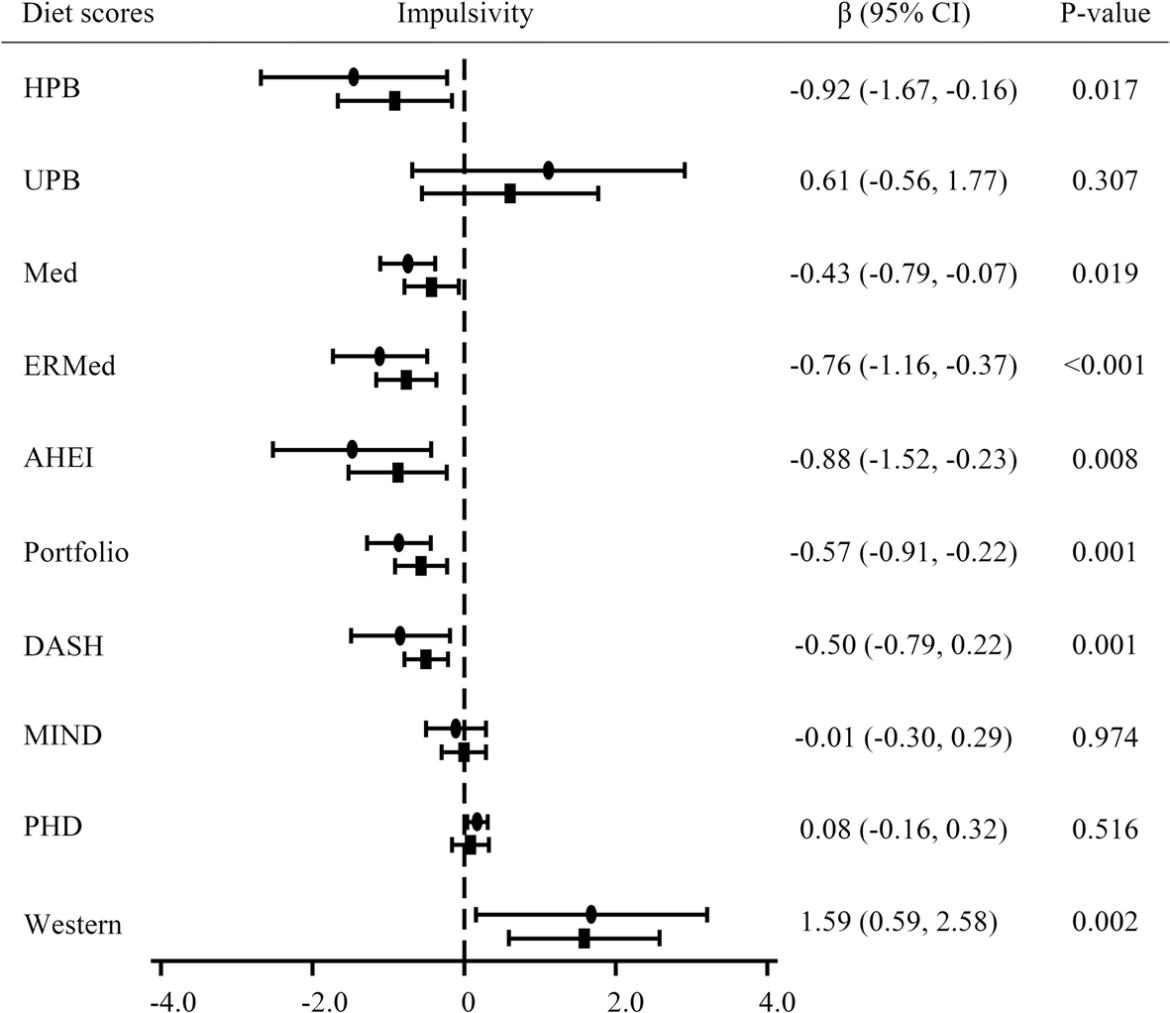
Longitudinal associations between impulsivity and dietary pattern scores (*n* = 438). Abbreviations: HPB, Healthy Plant-Based diet; UPB, Unhealthy Plant-Based diet; Med, Mediterranean diet; ERMed, Energy-Restricted Mediterranean diet, AHEI, Alternative Healthy Eating Index; Portfolio, Portfolio diet; DASH, Dietary Approaches to Stop Hypertension; MIND, Mediterranean-DASH Diet Intervention for Neurodegenerative Delay; PHD, Planetary Health Diet; Western, Western diet. Impulsivity was assessed using the UPPS-P Impulsive Behaviour Scale. Linear mixed models were used to assess associations and Beta coefficients were multiplied by 100, with robust variance estimators to account for intracluster correlations. Only model 2 beta coefficients and *p*-values were shown at the right of the figure. Circle dots referred model 1: associations were adjusted by sex, age (years) and intervention group at baseline. Square dots referred model 2: associations were further adjusted by education level (primary school or less; higher school or college), civil status (single, divorced, separated or widower; married) and smoking status (never smoked; former or current smoker) at baseline, whereas physical activity (MET min/week), body mass index (kg/m.^2^), alcohol intake (g/d, adding the quadratic term), and energy intake (kcal/d) at each time-point. All significant associations remained significant after Benjamini–Hochberg correction in both model 1 and model 2

All participants provided written informed consent, and the ethical committees of all participating institutions approved the study protocol and procedures.

### Impulsivity

Impulsivity was assessed at baseline, 1-year and 3-years of follow-up with the Impulsive Behavior Scale (UPPS-P) [15], validated for Spanish populations [25]. The UPPS-P is a 59-item self-reported questionnaire using a 4-point Likert scale (from 1 = “agree” to 4 = “disagree strongly”), which measures impulsivity traits by assessing 5 factors of impulsivity-related pathways: negative urgency, (lack of) perseverance, (lack of) premeditation, sensation seeking, and positive urgency. These 5-related impulsivity factors were obtained by combining their respective items, and the total UPPS-P score was obtained by summing all the unweighted UPPS-P items. Higher UPPS-P scores indicate higher levels of impulsivity. The α Cronbach values for the total score were 0.91 at baseline, 0.93 at 1-year of follow-up and 0.94 at 3-year of follow-up.

Although impulsivity trait is relatively stable over time, in our study it was preferred to assess impulsivity trait as a time-varying variable rather than solely at baseline in order to account for possible minimal variability, as for example impulsivity has been seen to change with increasing age and other factors [14], as well as to give more robustness to the analyses assessing impulsivity trait in more than one time-point.

### Covariates

Sex, age, level of education, civil status and smoking status were obtained at baseline from self-reported questionnaires administered by trained staff in face-to-face interviews. Physical activity, BMI, alcohol intake and energy intake were obtained at baseline, 1-year and 3-years of follow-up. Physical activity was evaluated using the short validated version of the Minnesota Leisure-Time Physical Activity Questionnaire [26]. Weight and height were measured by duplicate by trained staff using calibrated scales and wall-mounted stadiometers, respectively. BMI was calculated using the mean values of total body weight and height. Dietary intake was assessed via face-to-face interviews using the validated, semi-quantitative 143-item Food-Frequency Questionnaire (FFQ) [27, 28], and total energy and alcohol intake were obtained from the FFQs [27].

### Dietary pattern scores

The FFQ collected information on portion sizes and nine consumption frequencies (from “never or almost never” to “ ≥ 6 times/day”) for each assessed food item consumed over the previous year. Energy and nutrient intakes were obtained using data from the Spanish food composition database and by multiplying the frequency by the portion size, accounting for the duration of the period considered [29].

A total of 10 dietary pattern adherence scores were calculated. Eight dietary scores (Healthy Plant-Based diet, Unhealthy Plant-Based diet, Alternative Healthy Eating Index, Portfolio diet, Dietary Approaches to Stop Hypertension [DASH], Mediterranean-DASH diet Intervention for Neurodegenerative Delay [MIND], Planetary Health Diet, and Western diet) were determined based on data obtained from the 143-FFQ. Another score (the Energy-Restricted Mediterranean diet) was obtained from a 17-item Mediterranean diet questionnaire, which was assessed via face-to-face interviews by trained staff. The remaining diet score (the Mediterranean diet, without energy restriction specified) was obtained using data from the 143-FFQ as well as from equivalent items in the 17-point Mediterranean diet questionnaire.

Plant-based diets, characterized by higher plant food consumption than animal foods, are associated with a favorable cardiovascular disease risk [30]. In the present report, both the Healthy and Unhealthy Plant-Based dietary patterns were determined using the respective diet scores, with adherence scores ranging from 18 to 90 points [31]. Mediterranean diets have been associated with multiple health benefits, in particular cardiovascular health [32]. Adherence to an energy-restricted Mediterranean diet was evaluated by the 17-item Mediterranean diet questionnaire, with a score ranging from 0 to 17 points [33]. Adherence to the Mediterranean diet, without energy restriction, was determined based on the validated 14-item Mediterranean diet questionnaire (also called MEDAS), with the possible score ranging from 0 to 14 points [34, 35]. The 2010- Alternative Healthy Eating Index is a dietary pattern which follows the Dietary Guidelines for Americans and includes dietary factors involved in the development of chronic disease, with a possible score ranging from 0 to 110 points [36]. The Portfolio diet is a plant-based dietary pattern that combines recognized cholesterol-lowering foods, with an adherence score ranging from 6 to 30 points [37]. The DASH diet is a dietary pattern that was developed to reduce hypertension, and possible scores could range from 0 to 40 points [38]. The MIND diet is a diet tailored to protect against cognitive decline, and adherence scores could range from 0 to 15 points [39]. The Planetary Healthy Diet score defined by the EAT-Lancet commission is an ecological dietary pattern based on sustainable food choices, with possible adherence scores ranging from 0 to 14 points [40]. A Western diet presents high consumption of red meat and fast or fried food and low food intake of fruits, vegetables and fish [41, 42], being this dietary pattern associated with different health issues [42]. Adherence of these 10 dietary scores were evaluated in order to broadly assess possible differences between impulsivity and several dietary patterns, and for the sake of testing robustness (i.e., to determine if all healthy dietary scores or patterns tend to be negatively associated and all unhealthy dietary patterns are positively related). Supplemental Table 1 provides a description of the method performed to obtain the Western diet score, with a possible score ranging from 12 to 60 points [see Additional file 1]. For all aforementioned dietary patterns evaluated, higher scores indicate higher adherence to their respective dietary pattern.

**Table 1:** Baseline participant characteristics (*n* = 462)

General characteristics	Values
Age (years)	65.3 ± 4.7
Sex (women)	238 (51.5)
Intervention group	228 (49.3)
Education level	
Primary school or less	251 (54.3)
High school or college	211 (45.7)
Civil status	
Single, divorced, separated or widower	98 (21.2)
Married	364 (78.8)
Smoking status	
Never smoked	231 (50.0)
Former or current smoker	231 (50.0)
Physical activity (MET min/week)	2343 ± 2004
Body Mass Index (kg/m^2^)	32.5 ± 3.4
Psychological assessment (Range)	
UPPS-P Impulsive Behavior Scale (0–236 points)	108.1 ± 22.6
Energy intake and food groups consumption	
Energy intake (kcal/d)	2329 ± 475
Fruit (g/d)	340.2 ± 174.0
Vegetables (g/d)	344.2 ± 136.0
Legumes (g/d)	19.0 ± 9.82
Nuts (g/d)	13.5 ± 13.7
Extra virgin olive oil (g/d)	32.7 ± 19.9
Grains (g/d)	145.4 ± 69.0
Fish (g/d)	109.8 ± 43.1
Meat (g/d)	156.2 ± 56.3
Dairy products (g/d)	323.7 ± 179.7
Alcohol (g/d)	8.7 ± 11.3
Dietary pattern scores (Range)	
Healthy Plant-Based diet (18–90 points)	56.6 ± 7.6
Unhealthy Plant-Based diet (18–90 points)	57.3 ± 6.5
Mediterranean diet (0–14 points)	8.0 ± 1.8
Energy-restricted Mediterranean diet (0–17 points)	7.9 ± 2.5
Alternative Healthy Eating Index (0–110 points)	65.3 ± 8.6
Portfolio diet (6–30 points)	16.7 ± 4.0
DASH diet (0–40 points)	23.8 ± 5.2
MIND diet (0–15 points)	9.2 ± 1.2
Planetary Health Diet (0–14 points)	9.2 ± 1.5
Western diet (12–60 points)	28.9 ± 5.6

*Abbreviations*: *DASH* Dietary Approaches to Stop Hypertension, *MIND* Mediterranean-DASH Diet Intervention for Neurodegenerative Delay

Data are expressed as n (%) for categorical variables and mean ± SD for quantitative variables

The UPPS-P was measured only in 349 participants

### Statistical analysis

For the current analyses, the PREDIMED-Plus-Cognition study database updated in September 2021 was used.

Baseline participant characteristics are presented as numbers and percentages for categorical variables and mean ± standard deviation for quantitative variables. Linear mixed models were performed to assess longitudinal associations between the impulsivity trait as the exposure variable and dietary pattern scores as the outcomes, both measured as continuum time-varying variables. Associations between UPPS-P subscales and dietary patterns were also assessed using linear mixed models. Random effects were hierarchically established by center and by members sharing the same household unit (*n* = 418), respectively. The random intercept was performed for each participant and the random slope was performed considering baseline, 1-year and 3-years of follow-up data. Linear mixed models handle missing data accounting for the fact that repeated measures for each participant are intracorrelated, in the present study this meant including participants and using data when information from at least one of the three time-points was available. Two models were fitted to adjust linear mixed models. Model 1 was adjusted by sex, age (years) and intervention group. Model 2 was further adjusted by education level (primary school or less; high school or college), civil status (single, divorced, separated or widower; married) and smoking status (never smoked; former or current smoker) at baseline, whereas physical activity (MET min/week), BMI (kg/m^2^), alcohol intake (g/d, adding the quadratic term) and energy intake (kcal/d) were adjusted including data at each time-point.

Interaction analyses between impulsivity and sex, age and intervention group were performed by comparing the model with and without the interaction product using the likelihood ratio test. A sensitivity analysis was conducted to test the longitudinal associations between impulsivity and dietary patterns by intervention group. We also analyzed longitudinal associations between baseline UPPS-P score and 3-year adherence to healthy and unhealthy dietary patterns.

As the range of UPPS-P is large (0–236 points), all beta coefficients were multiplied by 100 hundred and robust variance estimators were used in all models to account for intracluster correlations. The data was analyzed using the Stata-14 software program (StataCorp). Statistical significance was set using the Benjamini–Hochberg false discovery rate correction procedure [43] at a Q-value < 0.05.

## Results

### Descriptive results

Table 1 shows baseline characteristics of the studied population (*n* = 462). The mean age was 65.3 ± 4.7 years and 51.5% were female. Approximately, a half of the participants had at least the primary school educational level, three quarters were married, and a half had never smoked. A total of 28.1% of the participants presented overweight (BMI < 30 kg/m^2^), with the rest having obesity (BMI ≥ 30 kg/m^2^). At baseline, the mean ± SD, minimum and maximum score for UPPS-P were 108.2 ± 22.7, 71 and 196 points, respectively. In regard to the UPPS-P scores at the 1- and 3-year follow-up the mean ± SD, minimum and maximum were 109.2 ± 24.5, 71 and 207, and 106.8 ± 24.1, 68 and 199, respectively. Baseline energy intake, food group consumption, and the 10 dietary pattern scores evaluated as mean ± SD are shown in Table 1.

For longitudinal analyses, UPPS-P measurement was conducted in 438 participants in which dietary assessments were conducted in all the study population.

### Impulsivity and dietary pattern scores

Figure 1 shows the longitudinal associations between impulsivity and adherence to diet scores over the 3 years of follow-up. Higher impulsivity values were associated with less adherence to the Healthy Plant-Based diet (β = -1.46; CI 95% [-2.68, -0.23]), Mediterranean diet (β = -0.74; CI 95% [-1.11, -0.38]), Energy-Restricted Mediterranean diet (β = -1.11; CI 95% [-1.73, -0.49]), Alternative Healthy Eating Index (β = -1.48; CI 95% [-2.52, -0.44]), Portfolio diet (β = -0.86; CI 95% [-1.28, -0.44]), and DASH diet (β = -0.84; CI 95% [-1.50, -0.19]) scores over 3 years of follow-up, and contrary, with higher adherence to the Western diet score (β = 1.68; CI 95% [0.15, 3.21]) over time, in model 1. These associations remained significant in the fully adjusted model. No significant associations were found in the case of the Unhealthy Plant-Based diets, MIND diet, and Planetary Health Diet. Supplemental Fig. 2 shows longitudinal associations between UPPS-P subscales and adherence to dietary pattern scores over time [see Additional file 1].

**Fig. 2:**
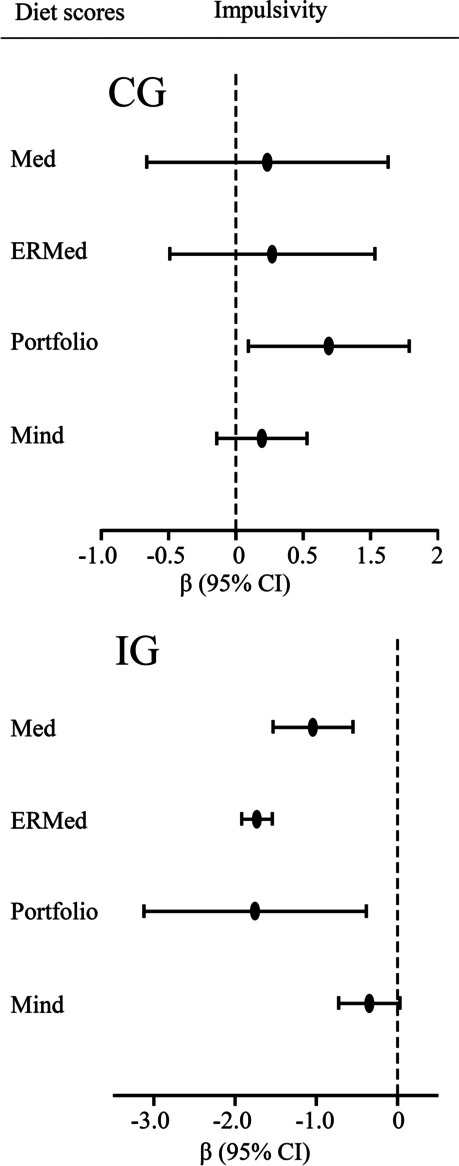
Interactions in longitudinal associations between impulsivity and dietary pattern scores by intervention group (*n* = 438). Abbreviations: CG, Control group; IG, Intervention group; Med, Mediterranean diet, ERMed, Energy-Restricted Mediterranean diet; Portfolio, Portfolio diet; MIND, Mediterranean-DASH Diet Intervention for Neurodegenerative Delay. Impulsivity was assessed using the UPPS-P Impulsive Behaviour Scale. Linear mixed models were used to assess associations and Beta coefficients were multiplied by 100, with robust variance estimators to account for intracluster correlations. Only significant interactions by intervention group were shown. Associations were adjusted by sex, age (years), intervention group, education level (primary school or less; higher school or college), civil status (single, divorced, separated or widower; married) and smoking status (never smoked; former or current smoker) at baseline, whereas physical activity (MET min/week), body mass index (kg/m.^2^), alcohol intake (g/d, adding the quadratic term), and energy intake (kcal/d) were considered using data at each time-point. Unless in the control group for Portfolio diet, all significant associations remained significant after Benjamini–Hochberg correction. CG, *n* = 219; IG, *n* = 219

There were no significant interactions by sex and age between impulsivity and dietary pattern scores (data not shown). Significant interactions by intervention or control group were found between impulsivity and adherence to the Mediterranean diet, Energy-Restricted Mediterranean diet, Portfolio diet, and MIND diet adherence scores over time (Fig. 2).

A sensitivity analysis was performed due to several significant interactions being displayed by the PREDIMED-Plus-Cognition trial intervention or control group between impulsivity and adherence to the assessed dietary patterns over 3 years of follow-up. This sensitivity analysis indicated a positive association between impulsivity and the Western diet score for the control group, whereas impulsivity was associated with lower adherence to the Mediterranean diet, Energy-Restricted Mediterranean diet, Alternative Healthy Eating Index, Portfolio diet, and DASH diet scores over time for the intervention group (Supplemental Table 2) [see Additional file 1]. We further analyzed longitudinal associations between baseline UPPS-P score and adherence to dietary patterns over 3 years of follow-up (data not shown), and the results followed similar trends although only the adherence to the Western diet remained statistically significant (β = 2.45; CI 95% [0.94, 3.96]).

## Discussion

To the best of our knowledge, the present study is the first to investigate longitudinal associations between impulsivity and adherence to various dietary patterns over 3 years of follow-up, while also accounting for several confounders. Our main finding showed that impulsivity was negatively associated with several healthy diet scores and positively related with adherence to an unhealthy dietary pattern in a Mediterranean population with overweight or obesity and metabolic syndrome participating in a lifestyle intervention program with a Mediterranean diet.

Limited research has been conducted studying relationships between impulsivity and dietary patterns. The NutriNet-Santé cohort has assessed cross-sectional associations between impulsivity and the representative score of the French Nutrition Guideline in an adult population, finding an inverse relationship [5]. In line with this study, our work also found in an older population that higher impulsivity levels were related with less adherence over 3 years of follow-up to most of the dietary scores assessed that have been associated with multiple health benefits [44]. Furthermore, impulsivity trait were positively associated with greater increases in the adherence to a Western diet style over time, a typical food pattern linked with an increased risk of chronic diseases [42]. However, a relationship between impulsivity trait and adherence to the MIND diet was not found. A possible explanation could be that the MIND diet is more permissive with red meat consumption, includes cheese consumption as a component of the score, and does not consider the consumption of fruits, aside from berries, in contrast to other healthy diet scores reflecting significant negative associations. In addition, impulsivity and adherence to the Planetary Healthy Index over time did not show a relationship either. In comparison with the diet scores displaying significant inverse associations, the Planetary Healthy Index allows for low-moderate but sustainable consumption of foods often considered to be more palatable, such as starchy vegetables, dairy foods, several kinds of protein sources (e.g., red meat or eggs), saturated oils, and added sugars, similar to the MIND diet. This leniency in food quantities and consumption of often more palatable food groups may explain why a non-significant relationship was observed between impulsivity and the MIND diet and the Planetary Healthy Diet, instead of a significant inverse association.

In our study, relationships between impulsivity and adherence to unhealthy dietary patterns over 3 years of follow-up showed discrepancies in their findings depending on the food group components of the diet scores assessed. Some evidence was found indicating positive cross-sectional relationships between impulsivity trait and the Western pattern style [19], but it is unknown for other non-healthy dietary patterns as those based in plant foods. In the present study, impulsivity showed a non-significant positive association with adherence to the Unhealthy Plant-Based diet over 3 years of follow-up, whereas a positive relationship was observed with the Western diet. This latter association demonstrated that participants with heightened impulsivity tend to easily follow a diet including a lower adherence to the consumption of healthy food groups (such as whole grains, fruits, vegetables, fish, nuts and legumes) and a higher adherence to the consumption of unhealthy food groups (such as refined grains, fast foods, red and processed meats, butters, sugar drinks, sweets and desserts) over time. Nevertheless, when impulsivity were assessed in relation to adherence to a food pattern representing a non-healthy plant-based diet, no association was shown. The Unhealthy Plant-Based diet considers animal food sources as a negative component, and this may be an explanation for the non-significance seen with impulsivity levels compared to adherence to the Western style diet over 3 years of follow-up.

Impulsivity is characterized by a tendency to act rashly under extreme positive or negative emotions, a lack of perseverance and premeditation, and the search for experiences and feelings [12, 15]. These attributes may predispose an individual to have greater difficulties in following a healthy dietary pattern that could be perceived as being less palatable or presents with restrictions to food groups which are socially commonly consumed. Furthermore, impulsivity is closely related to the reward system and food addiction [16, 18], and palatable foods have been suggested to affect the reward system in the same way as other kind of addictions such as alcoholism or drug abuse [45]. In addition, situational cues could induce more extreme behaviors in the spectrum of trait impulsivity [9]. For example, the power of heavy marketing has been observed to have an effect on prime automatic eating behaviors based on food advertisements, incrementing unhealthy food choices and the urge to eat [17, 46].

Consequently, our results demonstrated that higher impulsivity levels were more likely to be associated with a greater difficulty in adhering over 3 years of follow-up to healthy dietary patterns that have been shown to be useful in the prevention of cardiovascular-related problems or cancer [30, 32, 36, 37, 39, 44]. At the same time, people with high impulsivity levels tend to easily adhere to the unhealthy Western diet over time, a food pattern largely associated with different chronic diseases and health related conditions [42]. Therefore, food choices which may be driven by this psychological trait of impulsivity may play a role in increasing or reducing the risk of nutrition-related non-communicative diseases.

Our results showed few significant associations between negative urgency, positive urgency, sensation seeking, and perseverance UPPS-P subscales with adherence to some dietary patterns over 3 years of follow-up. This fact could be explained due to impulsivity being a multidimensional construct [15], suggesting that this psychological trait, as a composite, presents stronger associations with dietary patterns than specific impulsivity-related pathways.

Additionally, when impulsivity trait was only assessed at baseline, similar trends were shown for all dietary patterns as when we take into account impulsivity trait as time-varying variable, but only a significant positive association was found between impulsivity trait and adherence to the Western diet over time. These differences could be explained by using impulsivity as time-varying variable gives more robustness to our analyses. Moreover, these results could also manifest that little variations in impulsivity trait may occur over time leading to modifications in behaviors as it relates to adherence to dietary patterns.

In individuals not following an intervention program, we hypothesize that the same results would be found, as some research has shown cross-sectional positive associations between impulsivity trait and adherence to the healthy dietary French score in a total population of 51,043 participants.

Interestingly, interactions by PREDIMED-Plus intervention group were seen between impulsivity and adherence to some of the diet scores assessed over time. In comparison with participants in the control group, those in the intervention group showed significant negative associations between impulsivity and adherence to the Mediterranean diets and the Portfolio diet over 3 years of follow-up. Personality traits, including the psychological trait of impulsivity, might influence decisions as to whether an immediate over delayed reward is chosen. This could lead to overconsumption influenced by motivations and subsequently the selection of available highly palatable and more convenient foods instead of other more healthful options [8, 16, 47, 48]. As such, participants with higher impulsivity could present with difficulties delaying unhealthy food choices instead of waiting to choose a healthier option promoted by the active intervention program, derived by a lack of perseverance and forethought, and possibly driven by an overactivation of their reward system [16–18]. The key point is that this relationship was not observed in the control group. This may be because these participants were not required to follow the more restrictive intervention program focused on weight reduction and promotion of physical activity with behavioral support. Hence, participants in the control group did not have these added potential stressors and psychological conditions with which participants with higher impulsivity may not be able to manage additional general nutritional recommendations. Thus, higher impulsivity in the context of an active intervention program might result in less adherence to general healthy dietary recommendations.

Impulsivity has been observed to be modified in mindfulness intervention programs in individuals with drug abuse disorders [49, 50]. Therefore, mindfulness interventions could help participants to better follow dietary recommendations made by dietitians in those participants with high impulsivity due to improved emotion regulation and reduction of their impulsivity traits lack of premeditation and positive urgency.

One of the most important strengths of our study is the assessment of impulsivity and multiple dietary patterns at different time-point (3 times during a 3-year period), enabling a relatively robust longitudinal analysis. In addition, our study evaluated the associations between impulsivity and adherence to several dietary patterns over 3 years of follow-up, including healthy and unhealthy dietary patterns, but also other dietary patterns considering nutritional as well as ecological and environmental considerations. Another strength is that our analyses were adjusted for several relevant confounders. Furthermore, the impulsivity construct indicated a strong internal consistency showed by the Cronbach’s alpha values in each time point assessed. And finally, this analysis was performed in a relatively large study population compared with other studies assessing relationships between impulsivity and dietary assessments. However, different study limitations deserve to be recognized. The first and more important limitation is the observational study design, limiting the ability to establish causal relationships. Moreover, our study was conducted in an older Mediterranean population with overweight or obesity and at high risk of cardiovascular disease, and therefore the results may not be able to be extrapolated to other populations. Finally, FFQs present with some limitations of reliability, however in our study a validated FFQ was utilized, reducing the possibility of these reliability issues.

## Conclusions

Results showed that higher impulsivity trait values were associated with lower adherence to healthy dietary patterns over 3 years of follow-up in a population at high cardiovascular risk following a lifestyle intervention. Moreover, no associations between impulsivity and adherence to both the unhealthy plant-based dietary pattern and the Planetary Healthy Diet were found over time, possibly due to their less restrictive nature with regard to animal-based food consumption in its conception. Furthermore, impulsivity was positively related with greater adherence to a typical unhealthy Western style diet over time. Therefore, the impulsivity trait may be a risk factor of health diseases via participants’ decision-making related to food consumption. In addition, our results suggest that impulsivity should be considered in case of lifestyle intervention programs aimed at modifying dietary patterns as impulsivity is an important behavioral trait that potentially influence the participants’ capacity to adhere to the intervention.

## Supplementary Information

**Additional file 1: Supplemental Table 1.** Western diet score. **Supplemental Table 2.** Longitudinal associations between impulsivity and dietary pattern scores by intervention group (*n*=438).

**Additional file 2.**

**Additional file 3.**

## Data Availability

There are restrictions on the availability of data for the PREDIMED-Plus trial, due to the signed consent agreements around data sharing, which only allow access to external researchers for studies following the project purposes. Requestors wishing to access the PREDIMED-Plus trial data used in this study can make a request to the PREDIMED-Plus trial Steering Committee chair: predimed_plus_scommitte@googlegroups.com. The request will then be passed to members of the PREDIMED-Plus Steering Committee for deliberation.

## Abbreviations

95%CI: 95% Confidence interval
AHEI: Alternative Healthy Eating Index
BMI: Body Mass Index
DASH: Dietary Approaches to Stop Hypertension
ERMed: Energy-Restricted Mediterranean diet
MIND: Mediterranean-DASH Diet Intervention for Neurodegenerative Delay
Med: Mediterranean diet
PHD: Planetary Health Diet
Portfolio: Portfolio diet
UPB: Unhealthy Plant-Based diet
UPPS-P: Urgency, Premeditation (lack of), Perseverance (lack of), Sensation Seeking, Positive Urgency, Impulsive Behavior Scale
Western: Western diet
