# Correction: Impulsivity is longitudinally associated with healthy and unhealthy dietary patterns in individuals with overweight or obesity and metabolic syndrome within the framework of the PREDIMED-Plus trial

**DOI:** 10.1186/s12966-022-01373-2

**Published:** 2022-11-23

**Authors:** Carlos Gómez-Martínez, Nancy Babio, Jordi Júlvez, Stephanie K. Nishi, Fernando Fernández-Aranda, Miguel Ángel Martínez-González, Aida Cuenca-Royo, Rebeca Fernández, Susana Jiménez-Murcia, Rafael de la Torre, Xavier Pintó, Mirjam Bloemendaal, Montse Fitó, Dolores Corella, Alejandro Arias, Jordi Salas-Salvadó

**Affiliations:** 1 Universitat Rovira i Virgili, Departament de Bioquímica i Biotecnologia, Unitat de Nutrició Humana, Reus, Spain; 2 Institut d’Investigació Sanitària Pere Virgili (IISPV), Hospital Universitari Sant Joan de Reus, Reus, Spain; 3 Centro de Investigación Biomédica en Red de Fisiopatología de la Obesidad y Nutrición (CIBEROBN), Instituto de Salud Carlos III (ISCIII), Madrid, Spain; 4 Institut d’Investigació Sanitària Pere Virgili (IISPV), Clinical and Epidemiological Neuroscience Group (NeuroÈpia), Reus, Spain; 5 Toronto 3D (Diet, Digestive Tract and Disease) Knowledge Synthesis and Clinical Trials Unit, Toronto, ON Canada; 6 Clinical Nutrition and Risk Factor Modification Centre, St. Michael’s Hospital, Unity Health Toronto, Toronto, ON Canada; 7 Department of Psychiatry, School of Medicine and Health Sciences, University Hospital Bellvitge-IDIBELL and Department of Clinical Sciences, University of Barcelona, Barcelona, Spain; 8 Department of Preventive Medicine and Public Health. IdISNA, University of Navarra, Pamplona, Spain; 9 Integrative Pharmacology and Systems Neurosciences Research Group, Neurosciences Research Program, Hospital del Mar Medical Research Institute (IMIM), Barcelona, Spain; 10 Department of Preventive Medicine, University of Valencia, Valencia, Spain; 11 Faculty of Experimental and Health Sciences, Universitat Pompeu Fabra (UPF), Barcelona, Spain; 12 Lipids and Vascular Risk Unit, Internal Medicine, Hospital Universitario de Bellvitge-IBIDELL, Hospitalet de Llobregat, Barcelona, Spain; 13 Universitat de Barcelona, Barcelona, Spain; 14 Department of Human Genetics, Donders Institute for Brain, Cognition and Behaviour, Radboud University Medical Center, Nijmegen, The Netherlands; 15 Department of Psychiatry, Donders Institute for Brain, Cognition and Behaviour, Radboud University Medical Center, Nijmegen, The Netherlands

**Correction to:Following publication of the original article** [1], the authors would like to correct reference 40 and the name of Planetary Health Diet (PHD).

The incorrect name of dietary pattern score is: Planetary Healthy Dietary Index (PHDI).

The correct name of dietary pattern score is: Planetary Health Diet (PHD).

The incorrect reference is:

Cacau LT, De Carli E, de Carvalho AM, Lotufo PA, Moreno LA, Bensenor IM, Marchioni DM. Development and validation of an index based on EAT-lancet recommendations: the planetary health diet index. Nutrients. 2021;13:1698. doi: 10.3390/nu13051698.

The correct reference is:

EAT-Lancet Commission. Healthy Diets. In: EAT-Lancet Commission Summary Report about Food Planet Health: Healthy Diets From Sustainable Food Systems. EAT-Lancet Commission. 2019. https:/eatforum.org/eat-lancet-commission/eat-lancet-commission-summary-report. Accessed 8 Oct 2021.

The original article [1] has been corrected.

## References

[1] GómezMartínez et al. Int J. Behav Nutr Phys Act (2022) Impulsivity is longitudinally associated with healthy and unhealthy dietary patterns in individuals with overweight or obesity and metabolic syndrome within the framework of the PREDIMEDPlus trial (2022) 19:101 10.1186/s12966-022-01335-8. 10.1186/s12966-022-01335-8 PMC9358907 35941632

