# Supplementary material for: Impulsivity is longitudinally associated with healthy and unhealthy dietary patterns in individuals with overweight or obesity and metabolic syndrome within the framework of the PREDIMED-Plus trial
Source: Int J Behav Nutr Phys Act. 2022 Aug 8;19:101. doi: 10.1186/s12966-022-01335-8 (PMC9358907; doi:10.1186/s12966-022-01335-8)
Supplement: Supplementary file 1 — Additional file 1: Supplemental Table 1. Western diet score. Supplemental Table 2. Longitudinal associations between impulsivity and dietary pattern scores by intervention group (n=438). [file 12966_2022_1335_MOESM1_ESM.docx]

| **Supplemental Table 1** Western diet score | |
| --- | --- |
| Food group | Food components |
| Low intake |  |
| Whole grains | Integral pasta; integral rice; integral bread; integral muesli |
| Fruits | Orange, grapefruit or mandarin; banana; apple or pear; strawberry; cherry or plum; peach; watermelon; melon; kiwi; grape |
| Vegetables | Chard or spinach; cabbage, cauliflower or broccoli; lettuce, endive or escarole; tomato; carrot or pumpkin; green bean; eggplant, zucchini or cucumber; pepper; asparagus; gazpacho; onion; garlic; boiled potato; other vegetables |
| Fish | White fish; blue fish; natural canned fish; oil canned fish |
| Nuts | Almond; pistachio; walnut; other nuts |
| Legumes | Lentil; bean; chickpea; pea or broad pea; |
| High intake |  |
| Refined grains | White pasta; white rice; white bread |
| Fast/fried foods | Pizza; croquette; fried potato; chips; snacks |
| Red and processed meats | Calf; pork; lamb; hamburger or meatball; bacon; liver; other animal entrails; serrano ham; York ham or cocked ham; processed meats |
| Butters | Butter; margarine; lard |
| High sugar drinks | High sugar carbonated beverages; bottling or canned fruit juices |
| Sweets and desserts | Cookies; chocolate cookies; cake; sponge cake; croissant; donut; cupcake; churros; chocolate; nougat; marzipan |

The amount of each food component was determined by considering the frequency of consumption multiplied by the standard serving amount.

Each food group was divided into quintiles: 1^st^ quintile = 1 point; 2^nd^ quintile = 2 points; 3^rd^ quintile = 3 points; 4^th^ quintile = 4 points; 5^th^ quintile = 5 points.

The total score for each low intake food group were inversed.

Western diet score was obtained following the formula: Western diet = refined grains + fast/fried foods + red and processed meats + butters + high sugar drinks + sweets and desserts + whole grains + fruits + vegetables + fish + nuts + legumes.

| **Supplemental Table 2** Longitudinal associations between impulsivity and dietary pattern scores by intervention group (n=438) | | | | | |
| --- | --- | --- | --- | --- | --- |
| Dietary pattern scores | Intervention group | | | | |
|  | Control (n=219) | |  | Intervention (n=219) | |
|  | β (95% CI) | P-value |  | β (95% CI) | P-value |
| Healthy Plant-Based diet | -0.57 (-2.53, 1.40) | 0.573 |  | -1.57 (-4.09, 0,96) | 0.223 |
| Unhealthy Plant-Based diet | 0.69 (-1.77, 3.14) | 0.584 |  | 0.19 (-1.86, 2.24) | 0.854 |
| Mediterranean diet | 0.23 (-0.66, 1.13) | 0.610 |  | -1.04 (-1.53, -0.55) | <0.001* |
| Energy-restricted Mediterranean diet | 0.27 (-0.49, 1.03) | 0.487 |  | -1.73 (-1.92, -1.54) | <0.001* |
| Alternative Healthy Eating Index | 3.00 (-1.27, 1.87) | 0.708 |  | -2.28 (-3.03, -1.54) | <0.001* |
| Portfolio diet | 0.69 (0.09, 1.29) | 0.023 |  | -1.75 (-3.12, -0.38) | 0.012* |
| DASH diet | -0.20 (-1.26, 0.86) | 0.708 |  | -0.90 (-1.57, -0.23) | 0.009* |
| MIND diet | 0.19 (-0.14, 0.53) | 0.259 |  | -0.35 (-0.72, 0.03) | 0.071 |
| Planetary Health Diet | 0.29 (-0.25, 0.82) | 0.294 |  | -0.12 (-0.76, 0.52) | 0.713 |
| Western diet | 1.71 (0.47, 2.95) | 0.007* |  | 1.28 (-1.06, 3.63) | 0.282 |

Abbreviations: DASH, Dietary Approaches to Stop Hypertension; MIND, Mediterranean-DASH Diet Intervention for Neurodegenerative Delay

Linear mixed models were used to assess associations and beta coefficients were multiplied by 100, with robust variance estimators to account for intracluster correlations. Associations were adjusted for sex, age (years), education level (primary school or less; higher school or college), civil status (single, divorced, separated or widower; married) and smoking status (never smoked; former or current smoker) at baseline, and physical activity (MET min/week), body mass index (kg/m^2^), alcohol intake (g/d, adding the quadratic term), and energy intake (kcal/d) at each time-point.

* Significant associations after Benjamini-Hochberg correction.

**Supplemental Figure 1** Flow-chart of participants


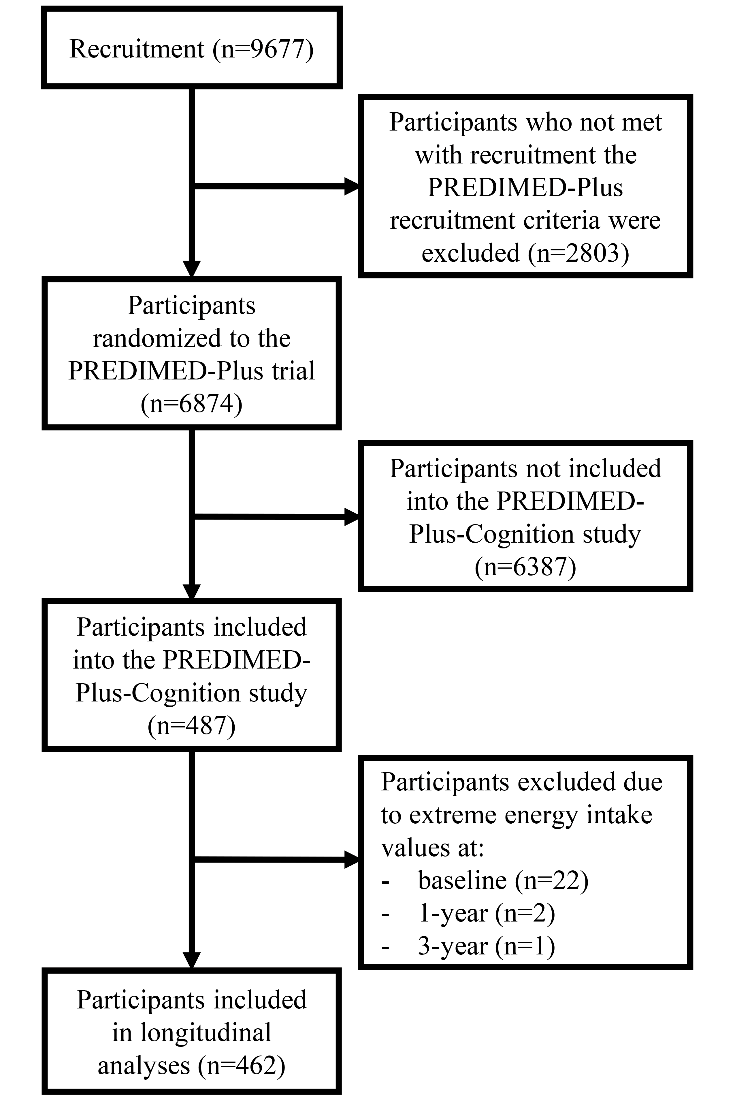


**Supplemental Figure 2** Longitudinal associations between UPPS-P subscales and dietary pattern scores (n=438)


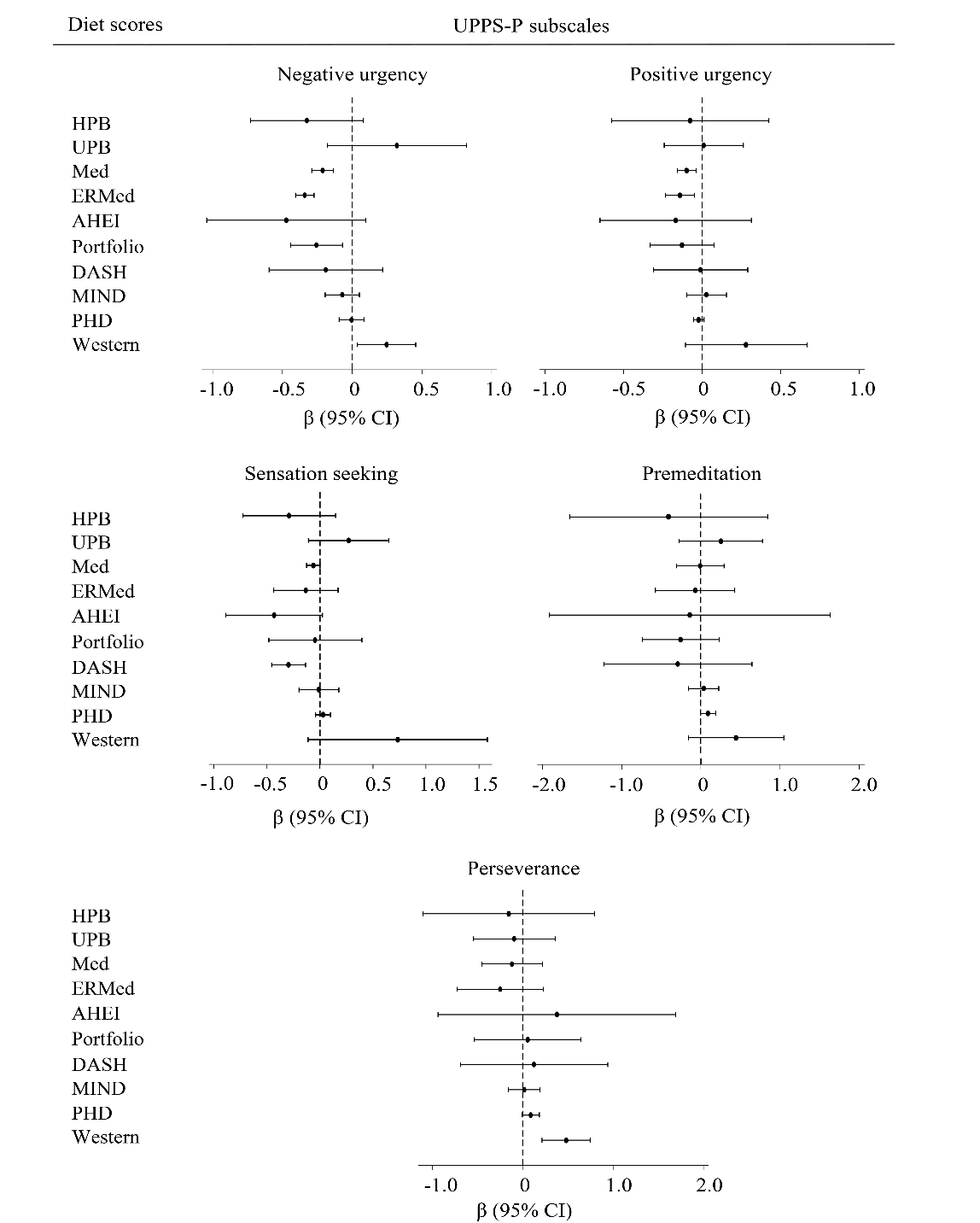


Abbreviations: HPB, Healthy Plant-Based diet; UPB, Unhealthy Plant-Based diet; Med, Mediterranean diet; ERMed, Energy-Restricted Mediterranean diet, AHEI, Alternative Healthy Eating Index; Portfolio, Portfolio diet; DASH, Dietary Approaches to Stop Hypertension; MIND, Mediterranean-DASH Diet Intervention for Neurodegenerative Delay; PHD, Planetary Health Diet; Western, Western diet.

Impulsivity was assessed using the UPPS-P Impulsive Behaviour Scale. Linear mixed models were used to assess associations and Beta coefficients were multiplied by 100, with robust variance estimators to account for intracluster correlations. Associations were adjusted by sex, age (years), intervention group, education level (primary school or less; higher school or college), civil status (single, divorced, separated or widower; married) and smoking status (never smoked; former or current smoker) at baseline, whereas physical activity (MET min/week), body mass index (kg/m^2^), alcohol intake (g/d, adding the quadratic term), and energy intake (kcal/d) at each time-point. Unless in negative urgency subscale for Western diet, all significant associations remained significant after Benjamini-Hochberg correction.
