# Supplementary figures and images for: Impulsivity is longitudinally associated with healthy and unhealthy dietary patterns in individuals with overweight or obesity and metabolic syndrome within the framework of the PREDIMED-Plus trial
Source: Int J Behav Nutr Phys Act. 2022 Aug 8;19:101. doi: 10.1186/s12966-022-01335-8 (PMC9358907; doi:10.1186/s12966-022-01335-8)

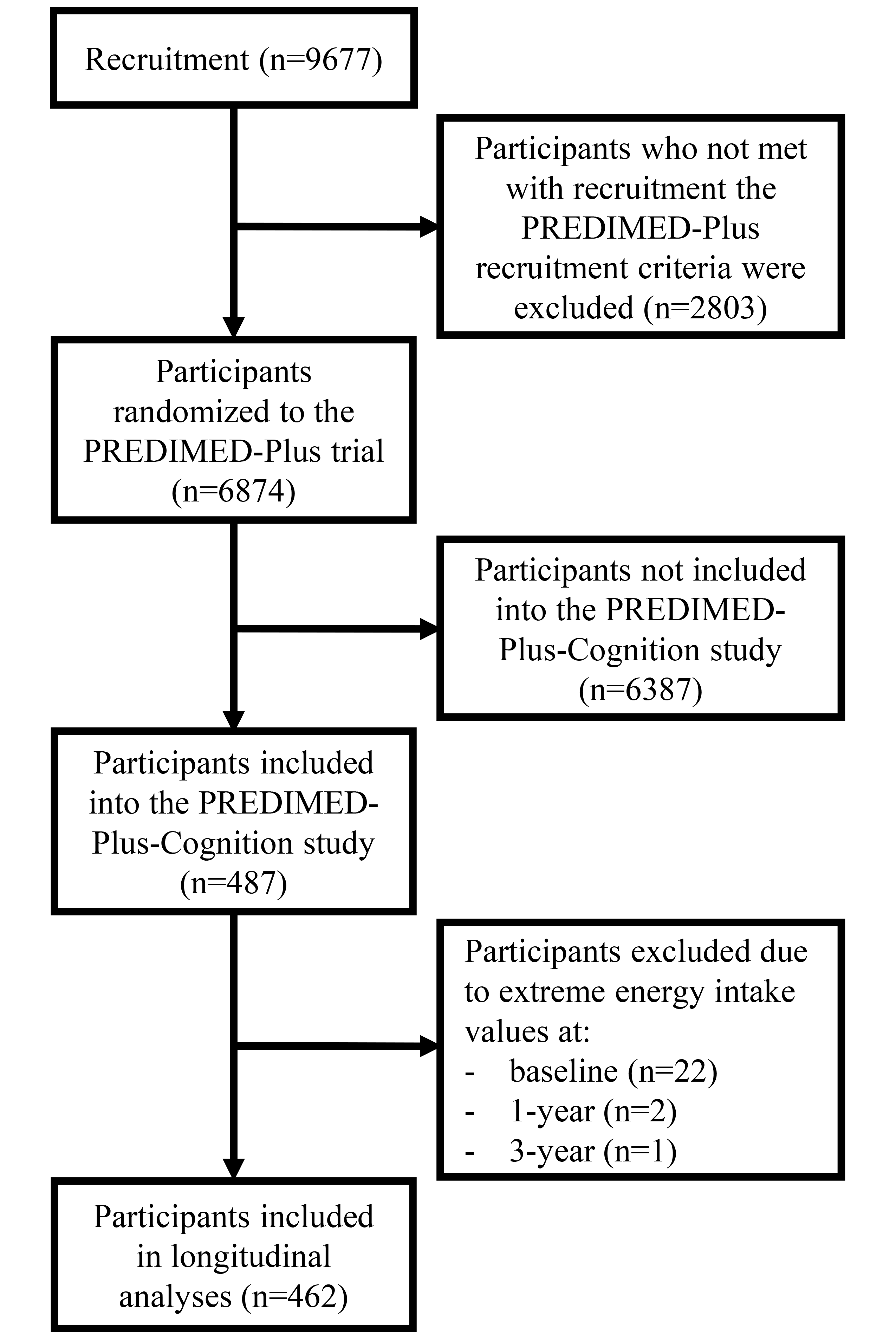

Supplement: Supplementary file 2 — Additional file 2. [file 12966_2022_1335_MOESM2_ESM.tif]

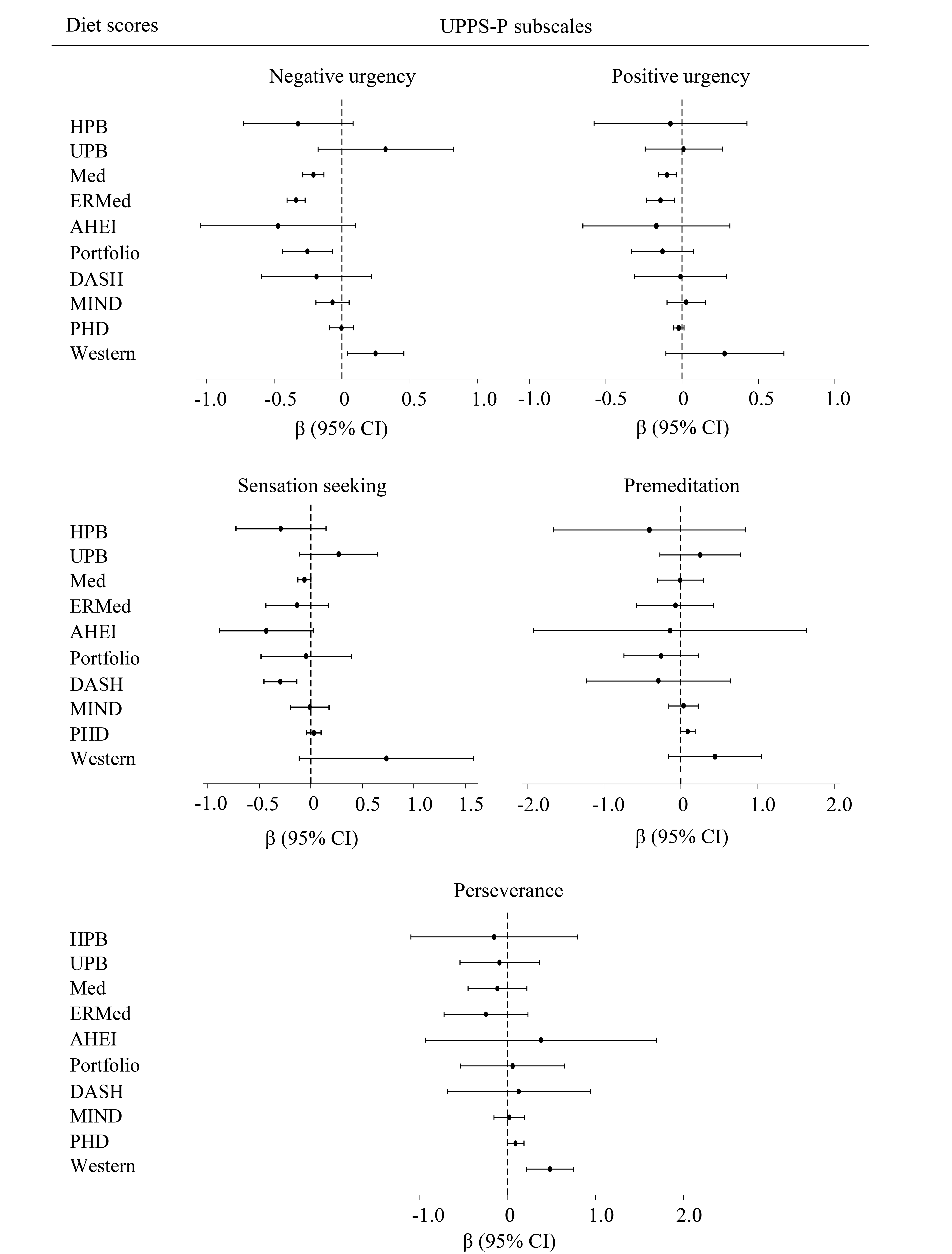

Supplement: Supplementary file 3 — Additional file 3. [file 12966_2022_1335_MOESM3_ESM.tif]
